# Transcriptomic Study on the Lungs of Broilers with Ascites Syndrome

**DOI:** 10.3390/ani13010175

**Published:** 2023-01-02

**Authors:** Dongqing Guo, Jian Zhang, Yufeng Han, Liang Cui, Huimin Wang, Keyao Wang, Peiqi Li, Ruiqiang Deng, Jie Kang, Zhibian Duan

**Affiliations:** College of Veterinary Medicine, Shanxi Agricultural University, Taigu 030801, China

**Keywords:** broiler ascites syndrome, lung, metabolic hypoxia, RNA-seq

## Abstract

**Simple Summary:**

This study used high-throughput sequencing to comprehensively study the lung changes of broilers with ascites syndrome (AS) at the transcriptome level and identified important pathways and significantly differentially expressed genes in lung tissue during the development of AS. Despite being extensively studied, its pathogenesis remains unclear, and the transcriptome of AS lung tissue in broilers has been underexplored. The study reveals the differential expression of lung genes and pathways in broilers, which is of great significance in studying the pathogenesis and prevention of AS. The genes identified in this study can be used as candidate genes for future lung studies of AS in broilers and provide a theoretical basis for studying AS in broilers.

**Abstract:**

Although broiler ascites syndrome (AS) has been extensively studied, its pathogenesis remains unclear. The lack of cardiopulmonary function in broilers causes relative hypoxia in the body; hence, the lung is the main target organ of AS. However, the transcriptome of AS lung tissue in broilers has not been studied. In this study, an AS model was successfully constructed, and lung tissues of three AS broilers and three healthy broilers were obtained for RNA sequencing (RNA-seq) and pathological observation. The results showed that 614 genes were up-regulated and 828 genes were down-regulated in the AS group compared with the normal group. Gene Ontology (GO) functional annotation revealed the following up-regulated genes: *FABP4*, *APLN*, *EIF2AK4*, *HMOX1*, *MMP9*, *THBS1*, *TLR4*, *BCL2*; and down-regulated genes: *APELA*, *FGF7*, *WNT5A*, *CDK6*, *IL7*, *IL7R*, *APLNR*. These genes have attracted much attention in cardiovascular diseases such as pulmonary hypertension. Kyoto Encyclopedia of Genes and Genomes (KEGG) enrichment analysis showed that multiple metabolic processes were enriched, indicating abnormal lung metabolism of AS in broilers. These findings elucidate the potential genes and signal pathways in the lungs of broilers with AS and provide a potential target for studying the pathogenesis and preventing AS.

## 1. Introduction

Ascites syndrome (AS) is also called broiler pulmonary hypertension syndrome. It is a common metabolic disorder, with main clinical symptoms including right heart hypertrophy and thinning, lung and liver congestion, edema, and hematoma [1]. Numerous studies have shown that hypoxia is the main cause of AS, and pulmonary hypertension is an important link in the pathogenesis of AS. Fast-growing broilers have a high metabolic rate, but the cardiopulmonary function of broilers is insufficient to support this and cannot provide enough oxygen to meet the metabolic demand of fast growth, resulting in relative hypoxia in the body. Hypoxia stimulates the formation of compensatory mechanisms in the cardiopulmonary system, including increased pulmonary vascular resistance [2], oxygen free radical production [3], and metabolic reprogramming [4], leading to pulmonary hypertension and further leading to the occurrence of AS. Although the pathological progression of AS has been studied for many years, the molecular mechanism of its occurrence and development has not been fully elucidated. Davoodi and Ehsani (2019) identified 24 genes that play a role in AS based on published studies, including *MPPK2*, *AT1*, *RhoGTPase*, *MC4R*, *CDH6*, *NOS3*, *HIF*-*1A*, *OSBL6*, *CCDC141*, *BMPR2*, *LEPR*, *AGTR1*, *UTS2D*, *5HT2b*, *SST*, *CHRD*, *TFRC*, *CDH13*, *ACVRL1*, *Arnt*, *ACE*, *ACVRL1*, *MEF2C*, and *HTR1A* [5]. A recent study found that *ALDH7A1*, *IRG1*, *GGT5*, *IGSF1*, *DHX58*, *USP36*, *TREML2*, *SPAG1*, *CD34*, and *PLEKHA7* are closely related to the pathogenesis of pulmonary artery remodeling in the progression of AS [6]. 

Both respiratory and circulatory systems are important for broiler development. The lack of any circulation between the lung, heart, and vascular system will lead to the above pathological cascade reaction, leading to the occurrence of AS in broilers [2]. Therefore, the lung is the main target organ of AS. It has been shown that in the lungs of rapidly growing broilers, all available blood vessels appear congested, demonstrating a very limited ability of the broiler lung to utilize key compensatory mechanisms. In addition, the lung plays an important role in many other processes, including the metabolism of endogenous and exogenous compounds, the regulation of water-fluid metabolism, and the production of a wide variety of signaling molecules (as a para-endocrine organ), including growth factors and cytokines [7,8]. The lung comprises various cell types (fibroblasts, airway smooth muscle cells, blood vessels, nerve cells, alveolar macrophages, mast cells, and dendritic cells) and the extracellular matrix. These cells constantly interact with extracellular components to achieve dynamic regulation of various functions. Any disruption of these interactions can lead to diseases (including asthma, chronic obstructive pulmonary disease (COPD), acute lung injury (ALI), pulmonary hypertension, and cancer) [7,9]. To date, several studies have been conducted on broilers with ascites, covering the liver [10], heart [11,12], and vasculature [13]. However, the transcriptome of lung tissue in broilers with AS has not been studied.

We used high-throughput sequencing (RNA-seq) to comprehensively study the lung changes of broilers with AS at the transcriptome level and identified important pathways and significantly differentially expressed genes in lung tissue during the development of AS. This study aims to reveal the differential expression of lung genes and pathways in broilers, which is of great significance for studying the pathogenesis and prevention of AS.

## 2. Materials and Methods

### 2.1. Animal Management and Construction of an Ascites Model

A total of 168 one-day-old male and female mixed (1:1) Arbor Acres commercial broilers were reared according to the routine method. At 8 days of age, they were randomly divided into the following two groups: normal group (N) and multifactor model group (C). There were 3 replicates in each group and 28 broilers in each replicate. The indoor temperature was 32–35 °C in the first week and decreased by 1–2 °C daily from 8 days of age to 25 °C at 11 days of age. After that, group N was fed routinely at a room temperature of 25 °C. In group C, the temperature was dropped to 9–11 °C daily, which remained unchanged until the end of the experiment. The susceptibility of ascites was increased by adding 3% lard and 4% fish meal to the basal diet per kg to increase the energy and fat in the basal diet, and 0.12% NaCl was added to the water in group C in the course of the experiment. Basic diet composition and nutrition level are shown in Table 1 and Table 2. All chickens had free access to food and water and were maintained at a light intensity for 40 W/20 m^2^ for 23 h and one hour in the dark. 

### 2.2. Sample Collection

At the age of 15, 25, and 35 days, the broilers’ feed intake and body weight in each group were measured, and the average body weight, feed conversion ratio (FCR), total mortality, and incidence of ascites were calculated. In the present study, only male broilers were selected for follow-up tests. Three broilers were randomly selected from each repetitive group for autopsy. In the model group, chickens with distention of the abdomen and a feeling of fluctuation were selected whenever possible and weighed after 8 h of fasting. Before dissection, blood from the chicken was collected from the subalar vein, and routine blood analysis was performed using an automatic blood analyzer (Shenzhen Pukang Electronic Co., Ltd., Shenzhen, China). The broilers were then slaughtered by jugular vein exsanguination, and their lungs and hearts were dissected. The two atria of the heart were cut along the coronary groove, excess fat and blood clots were removed, and the whole ventricle was accurately weighed. The right ventricle was then cut along the anterior and posterior longitudinal sulci and weighed accurately. Right ventricular weight and total ventricular weight were recorded to calculate the ascites cardiac index (AHI) using the following formula:

Ascites heart index (AHI) = right ventricular weight (RV)/total ventricular weight (TV)(1)

Then, the lung weight was accurately measured, and the lung organ coefficient was calculated (the ratio of lung wet weight to its body weight).

A part of the lung tissue was quickly frozen in liquid nitrogen and stored at −80 °C for transcriptome and quantitative real-time polymerase chain reaction (qRT-PCR) analysis. Fresh left lung tissue was fixed in 4% paraformaldehyde transversally along the hilum and dehydrated with graded concentrations of ethanol. The fixed lung tissue was embedded in paraffin. The embedded wax blocks were cut into 5 μm slices using a KD2508 paraffin slicer (Shanghai Yuejin Medical Equipment Co., Ltd.). The sections were stained using hematoxylin-eosin staining, and the lung tissue morphology was observed using a Leica DM4000B microscope (Wetzlar, Germany). Three normal and three broilers with AS were selected for the lung tissue transcriptome analysis. Broilers were selected on the basis of a yellow effusion in the abdominal cavity or pericardium, AHI > 0.29, and hematocrit (HCT) > 36% [10].

### 2.3. Total RNA Extraction, cDNA Library Construction, and RNA-seq

Total RNA was extracted from lung tissue (91 to 99 mg per sample, three samples per group) using TRIzol reagent (Invitrogen, Carlsbad, CA, USA). The concentration and purity of RNA were detected using a NanoDrop 2000 (Nanodrop Technologies, Wilmington, DE, USA). The integrity of RNA was determined using agarose gel electrophoresis, and the value of RNA integrity number (RIN) was determined using the Agilent_2100 bioanalyzer (Agilent Technologies, Santa Clara, CA, USA). The following was ensured for library construction: total RNA not less than 1 µg, the concentration of RNA not lower than 35 ng/μL, OD_260/280_ purity between 1.8 and 2.2, OD_260/230_ purity not less than 1.0, and RIN value not less than 7.0. The Illumina Truseq^TM^ RNA sample preparation kit method was used to construct the library in the sequencing experiment. The library was sequenced on the Illumina Novaseq 6000 platform (Meiji Technology Co., Ltd., Shanghai, China); high-quality clean data (reads) after quality control were compared with the reference genome *GRCg6a* (http://asia.ensembl.org/Gallus_gallus/Info/Index; GenBank: GCA_000002315.5; accessed on 27 March 2018). Sequence alignment analysis was performed using the TopHat2 software (Version v2.1.1) (http://tophat.cbcb.umd.edu/, accessed on 28 June 2020) to obtain mapped data (reads) for follow-up analysis. The quality of sequence alignment results was evaluated from the point of view of the sequence saturation, sequence coverage, read distribution in different regions, and different chromosome read distributions. The raw counts were analyzed using DESeq2 software (http://bioconductor.org/packages/stats/bioc/DESeq2/, accessed on 28 June 2020), based on the negative binomial distribution, and the *p*-value obtained by statistical tests was corrected using the Benjamini-Hochberg method (false discovery rate correction) to identify the differentially expressed genes among samples, and subsequently, to study the function of differentially expressed genes.

### 2.4. Gene Ontology Annotation and Kyoto Encyclopedia of Genes and Genomes (KEGG) Pathway Enrichment Analysis

Gene ontology (GO) enrichment analysis of the genes in the gene set was performed using GOATOOLS (https://github.com/tanghaibao/GOatools, accessed on 28 June 2020). The Kyoto Encyclopedia of Genes and Genomes (KEGG) is a large knowledge base for analyzing gene function, contact genome information, and functional information (https://cloud.majorbio.com/, accessed on 28 June 2020).

### 2.5. Quantitative Real-Time PCR (qRT-PCR)

The accuracy of the RNA-seq results was verified using real-time qPCR. Six genes were randomly selected and amplified using primers designed according to the mRNA sequence in the NCBI database (Table 3). The qPCR was performed using the 2 × T5 Fast qPCR Mix (Takara Biological Co., Ltd., Beijing, China) in a real-time thermal cycler (Wuhan Qingke Biotechnology Co., Ltd., Wuhan, China). The expression of each gene was evaluated using the 2^−ΔΔCt^ method [14,15].

### 2.6. Statistical Analysis

The qRT-PCR results were statistically analyzed using the GraphPad Prism software v.9.0. The SPSS17.0 software (SPSS Inc., Chicago, IL, USA) was used for two-factor variance analysis to analyze the significance of the main effect and interaction between treatment group and days. *p* < 0.05 is considered to be statistically significant.

## 3. Results

### 3.1. Routine Blood Parameters and Lung Pathology 

**Figure 1:**
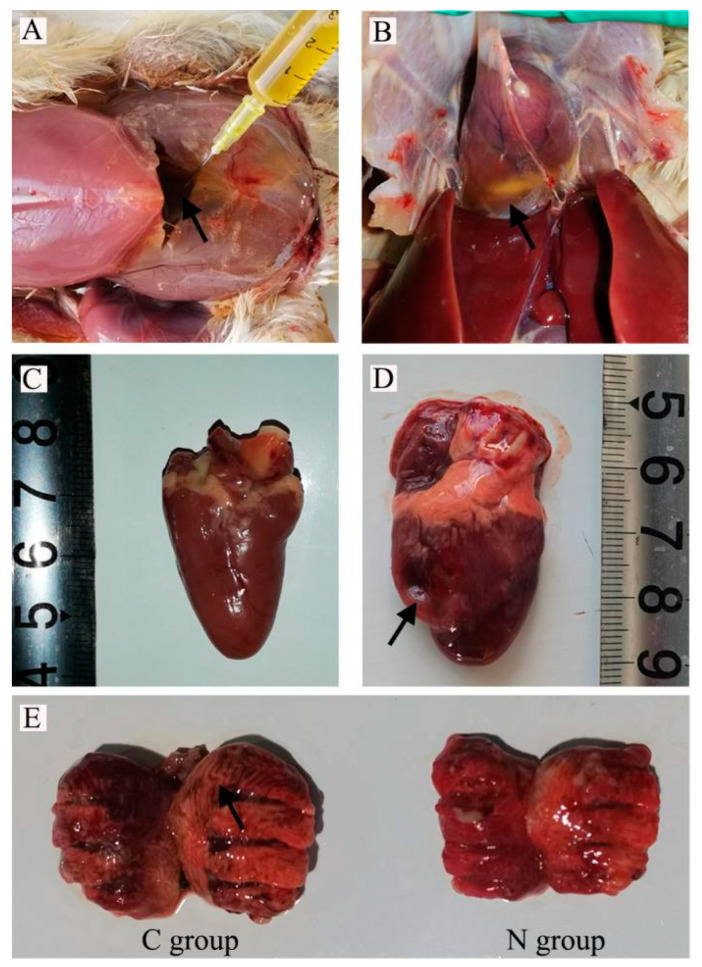
Dissection of the lesions associated with broiler ascites syndrome (arrow). (**A**) Removal of a large amount of yellow fluid accumulated in the abdominal cavity. (**B**) Obvious pericardial effusion. (**C**) Normal broiler heart. (**D**) Ascites syndrome broiler heart showing significantly increased heart volume, and the right ventricle dilated and thinned. (**E**) C group: ascites syndrome broiler enlarged lungs with gray and white striations in the interstitium. N group: normal broiler lungs.

The clinicopathological observations of group N and group C are shown in Figure 1. Compared with group N, AS broilers in group C showed abnormal breathing, abdominal swelling, and fluctuating tissue, with thinning, shining abdominal skin. The abdomen of AS broilers showed large amounts of yellow fluid and fibrin clots in the abdominal cavity (Figure 1A), clear pericardial effusion (Figure 1B), heart swelling, deformation, softness, marked dilatation and thinning of the right ventricle (Figure 1C,D), and lung congestion and edema, showing a speckled shape (Figure 1E). 

Multivariate analysis for AS parameters of broilers showed that (Table 4): (1) there were significant differences (*p* < 0.01) in average body weight, AHI, and pulmonary organ coefficient between group C and group N; (2) there were significant differences (*p* < 0.01) in average body weight and AHI among different ages; (3) two-factor treatment interaction between treatment and days indicated that it had a significant effect on average body weight and AHI (*p* < 0.01).

Multivariate analysis for blood routine index of broilers showed that (Table 5): (1) there were significant differences (*p* < 0.01) in total erythrocyte (RBC), hemoglobin (HB), hematocrit (HCT), white blood count (WBC) and lymphocyte (LYM) between group C and group N; (2) there were significant differences (*p* < 0.01) in RBC, PLT, and PCT among different ages; (3) two-factor treatment interaction between treatment and days indicated that it had a significant effect on RBC, HB, HCT, PLT, and PCT (*p* < 0.01).

**Figure 2:**
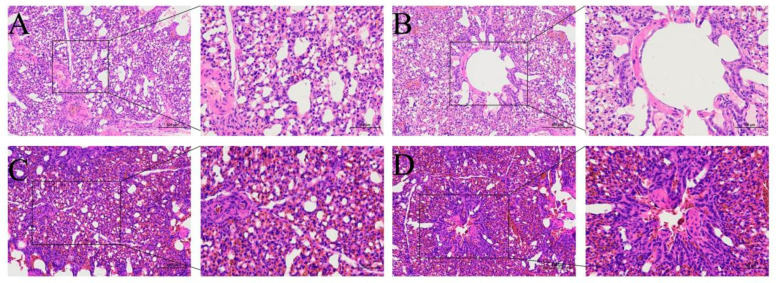
Hematoxylin and eosin-stained lung tissue (200×/400×). (**A**,**B**) The normal group. A: the pulmonary tissue structure and pulmonary vascular wall. B: the lung chamber. (**C**,**D**) The model group. (**C**) Pulmonary congestion and respiratory capillary atrophy. (**D**) Pulmonary atrial stenosis.

The pathological changes in lung tissue can be seen in Figure 2. In the N group, the pulmonary vascular wall of the lung tissue was normal, the respiratory capillary and lung chamber were clear, and no inflammatory cell infiltration was found (Figure 2A,B). Thickening of pulmonary capillaries, atrophy of respiratory capillaries, narrowing of lung atrium, and atrophy of surrounding smooth muscle could be seen in the lung tissue of broilers with ascites. In addition, pulmonary congestion, edema, and a small amount of lymphocyte infiltration were seen in chickens with ascites (Figure 2C,D).

### 3.2. RNA-seq Results

#### 3.2.1. Transcriptome Profiles

Six libraries prepared from RNA extracted from the lung tissue of broilers from groups N and C were sequenced by Illumina, and billions of original reads were produced. The quality control of the original sequencing data showed that the average error rate of the corresponding sequencing bases was less than 0.1%, Q20 was more than 85%, Q30 was more than 80%, and GC content was more than 50%. After comparing the clean data (reads) after quality control with the reference genome, the mapping rate was higher than 65% (C1, 92.44%; C2, 92.44%; C3, 92.67%; N1, 93.21%; N2, 93.24%; N3, 93.31%), all of which were located in the reference genome. The original data of this study in FASTQ format are stored in the Sequence Read Archive (SRA) database of the National Center for Biotechnology Information (NCBI) under the accession number PRJNA758269.

#### 3.2.2. Differential Gene Expression Analysis

**Figure 3:**
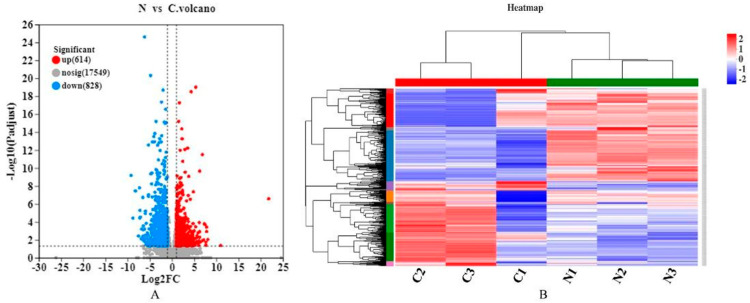
(**A**) Volcano map of differential expression. The red dots indicate significantly up-regulated genes, the green dots indicate significantly down-regulated genes, and the gray dots are nonsignificantly different genes. (**B**) Clustering heat map of differentially expressed genes in group N and group C. Red indicates high, and blue indicates low expression levels. N1, N2, and N3 indicate three parallel samples in the normal group. C1, C2, and C3 indicate three parallel samples in the multifactor ascites group.

A total of 1442 differentially expressed genes were identified in the lung tissues of groups N and C (*p*-adjust < 0.05, |log_2_FC| ≥ 1), of which 614 genes were up-regulated and 828 were down-regulated (Figure 3A). Table 6 lists the top 10 up-regulated and down-regulated genes. A cluster analysis shows the expression patterns of differentially expressed genes (Figure 3B).

#### 3.2.3. Gene Set Analysis

**Figure 4:**
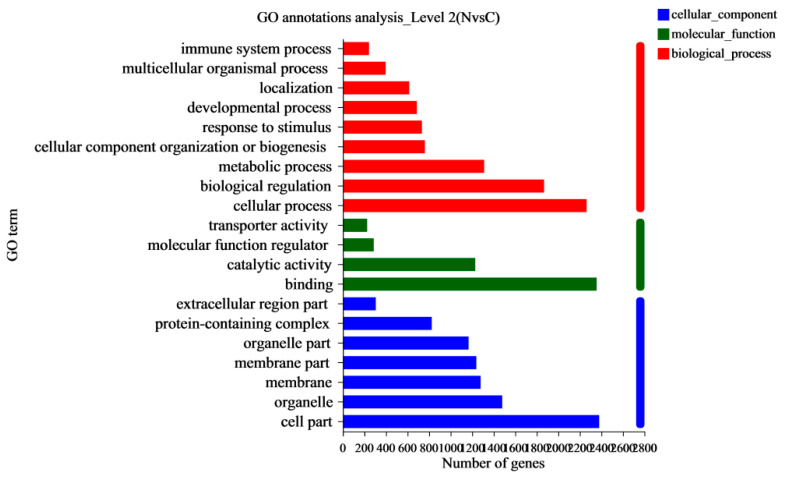
Genetic ontology analysis and classification statistics. The ordinate represents the second-level classification, and the abscissa represents the number of genes. The different colors represent molecular function, cellular components, and biological processes.

**Figure 5:**
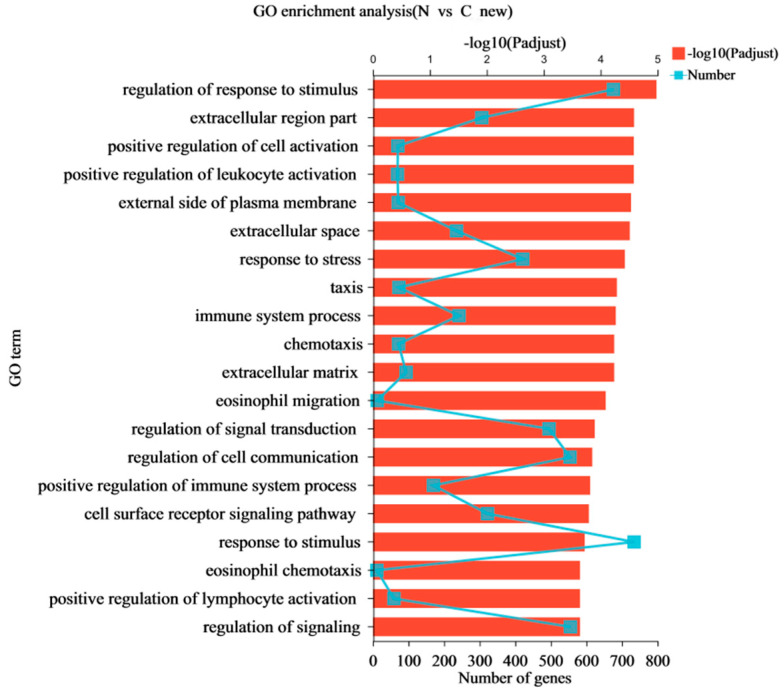
Up-regulated and down-regulated genes were obtained using Genetic Ontology enrichment analysis (*p* < 0.05). The *x*-axis at the bottom indicates the number of genes corresponding to the points on the broken line. The *x*-axis at the top indicates the significance of enrichment corresponding to the bar’s height.

**Figure 6:**
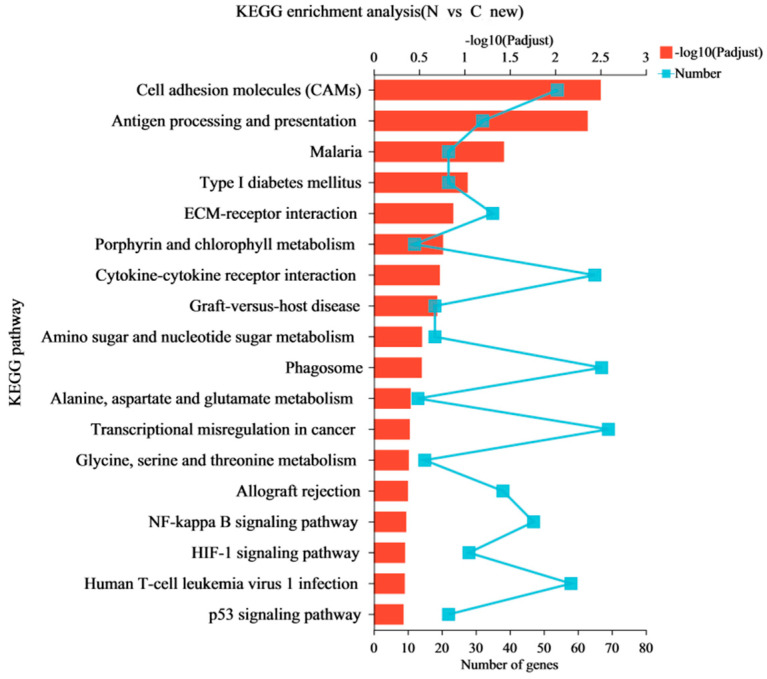
Up-regulated and down-regulated genes were obtained using KEGG enrichment analysis. The *x*-axis at the bottom indicates the number of genes corresponding to points on the broken line. The *x*-axis at the top indicates the significance of enrichment corresponding to the bar’s height.

Gene Ontology (GO) annotation assigned 1442 differentially expressed genes (DEGs) in GO terms to classify and annotate genes from the three aspects of molecular function, cell composition, and biological processes. The most abundant GO terms related to DEGs are shown in Figure 4. These DEGs mainly focus on biological processes and cell composition. In terms of biological process classification, GO terms with significant enrichment included “cellular process”, “biological regulation”, and “metabolic process”. In terms of cell composition, most DEGs were enriched in the GO terms of “cell part” and “organelle”. Catalytic activity DEGs were mainly classified into “binding” and “catalytic activity” in the molecular function categories. A further GO enrichment analysis was conducted (Figure 5). The most dominant GO process was “regulation of response to stimulus”, which included 676 DEGs, and the key DEGs are summarized in Table 7. A KEGG enrichment analysis (Figure 6) showed that of the top 20 key pathways, the most significantly enriched pathway was cell adhesion molecules (CAMs), which may be a response to endothelial dysfunction or vascular injury. In addition, it was enriched in the “HIF-1 signaling pathway”, which is a key pathway for developing pulmonary hypertension. Moreover, several metabolic processes, including “Amino sugar and nucleotide sugar metabolism”, “Alanine, aspartate and glutamate metabolism”, “Glycine, serine and threonine metabolism” and “NF-kappa B signaling pathway”, “p53 signaling pathway”, were enriched, which may be involved in the process of hypoxia-induced pulmonary arterial hypertension (Table 7). We searched the DEG functions of the most dominant GO-enriched processes and KEGG key pathways by reviewing the literature extensively. The up-regulated genes, *APLN*, *FABP4*, *EIF2AK4*, *HMOX1*, *MMP9*, *THBS1*, *TLR4*, and *BCL2* and down-regulated genes, *APELA*, *FGF7*, *WNT5A*, *CDK6*, *IL7*, *IL7R*, and *APLNR*, were found. These genes are of great interest in cardiovascular diseases such as pulmonary hypertension.

**Figure 7:**
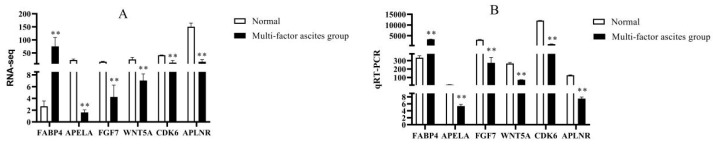
The qRT-PCR validation of RNA sequencing results. (**A**) RNA-seq results, (**B**) qRT-PCR results; ** indicates *p* < 0.01.

In order to verify the reproducibility and accuracy of the gene expression data obtained by RNA-Seq, we randomly selected six genes for qRT-PCR validation. The qRT-PCR results showed that *FABP4* was up-regulated in group C (*p* < 0.01); *APELA*, *FGF7*, *WNT5A*, *CDK6*, and *APLNR* were down-regulated in group C, compared to group N (*p* < 0.01) (Figure 7B), which was consistent with the results of the RNA-Seq (Figure 7A).

## 4. Discussion

The pathogenesis of AS in broilers may be due to insufficient circulation between the heart, lung, and vascular systems to meet the metabolic needs of broilers. To some extent, defects in one system can prevent the other parts from being fully compensated, thus triggering a pathological cascade that eventually leads to ascites [16]. The lung is an important target organ of AS [17]. Hasanpur et al. (2019) found that lung weight, as an indicator of respiratory capacity, was the most important factor affecting the sensitivity of broilers to AS [11]. It is consistent with the results of this study. Therefore, decreased respiratory capacity is the most likely factor to induce AS. In the current study, the average body weight of broilers in group C decreased, AHI increased, and AHI values more than 0.29. Compared with group N, the blood routine indexes increased, the levels of HCT and HB increased, and there was yellow fluid accumulation in the abdominal cavity and pericardium. Thus, the AS broiler model group was successfully established. In the current study, AHI (RV/TV) > 0.29, HCT, and HGB levels were increased compared with normal chickens, and there was yellow fluid accumulation in the abdominal cavity and pericardium. Hence, the AS broiler model group was successfully constructed. The lung tissue of broilers with ascites was congested, edematous, mottled, and slightly hard in texture, with grayish-white streaks in the interstitium (Figure 1a). The pathological changes in the lung were confirmed by the tissue sections. There was a tendency for the pulmonary vascular walls to thicken; the lung chamber was narrow, and there was respiratory capillary atrophy. Furthermore, there were pulmonary congestion, edema, and signs of inflammation in the lungs. 

In this study, RNA-Seq was performed on the lung tissues of the normal group (group N) and the AS model group (group C) to identify the differential expression of possible candidate genes and pathways in the lung tissues and to elucidate the pathological mechanism of AS in broilers. DEGs and different regulatory pathways that may be involved in the pathogenesis of AS were found in the GO enrichment and KEGG enrichment pathways. We found that “Cell adhesion molecules (CAMs)” and the HIF-1, NF-κB, and p53 signaling pathways were enriched in AS. It has been shown that endothelial cells overexpress adhesion molecules in response to stimuli, such as turbulent flow, dyslipidemia, or increased luminal pressure. The Refmir test has proven that increased blood pressure is associated with increased serum CAMs concentration and decreased circulating leukocyte ligands freely accessible by CAMs [18]. In the current study, the CAMs pathway was most significantly enriched, possibly in response to endothelial dysfunction or vascular injury. In addition, sequencing results in this study showed that the HIF-1 signaling pathway was very important in the occurrence and development of AS. In lung tissue, HIF-1 signaling orchestrates the body’s physiological response to oxygen. By participating in nitric oxide (NO) and the corresponding metabolic changes, the HIF signaling pathways increase pulmonary hypertension (pulmonary artery hypertension, PAH) vascular lesions [19]. NF-κB and p53 signaling pathways interact with each other to affect hypoxic PAH, thus playing a role in the occurrence and development of AS. Compared to wild-type mice, p53-knockout mice exhibit more severe PAH in response to chronic hypoxia [20]. Studies have found that TNF-activated NF-κB and p53 signaling pathways regulate the abnormal function of platelet mesenchymal stem cells, thereby improving hypoxic PAH [21]. In addition, studies have shown that persistent hypoxia can induce the accumulation of leukocytes and mesenchymal progenitor cells in pulmonary arteries, promoting the formation of a pulmonary artery-specific chronic inflammatory microenvironment [22]. The inflammatory mechanism of chemokine, fractalkine, and its receptor, Cx3CR1, in the lungs of patients with pulmonary hypertension may play a role in the natural development of pulmonary hypertension, proving the possibility of an inflammatory component in pulmonary hypertension [23]. 

The “HIF-1 signaling pathway” plays an important role in regulating the AS hypoxic stress response in broilers. The pathology of rapid development of AS is caused by hypoxia. Hypoxia-inducible factors (HIFs) are major regulators of angiogenesis, erythropoiesis, and metabolism regulation [24], and their expression is substantially increased in the lungs and heart of broilers with AS [25]. The HIF-1α subunit is a unique functional subunit of HIF-1, which is closely related to hypoxia. The ratio of lung volume to body weight of broilers is small, implying that the respiratory system cannot respond to the high oxygen demand of broilers, leading to hypoxia and increased oxidative stress [26]. HIF-1 is a key transcriptional factor of hypoxia-induced genes, which can initiate a vast hypoxic stress regulatory network under hypoxia.

Several pathways related to metabolic processes were enriched in this study, including amino sugar and nucleotide-sugar metabolism, alanine-aspartate-glutamate, and glycine-serine-threonine metabolism. A growing number of studies have identified a pervasive feature of metabolic and bioenergetic alterations in human and animal models of pulmonary hypertension, as metabolic abnormalities have been found in the lungs and hearts of patients, in animal models of this disease, and in cells derived from the lungs of patients. Stenmark et al. proposed that metabolic reprogramming is involved in the pathogenesis of PAH [4,27]. Metabolic reprogramming refers to the rearrangement of disordered metabolic pathways in the body in response to changes in the internal and external environment, i.e., the reintegration of metabolic systems to provide energy for cells [28]. Key features of PAH metabolism include shifts in glycolysis, increases in glutamine availability and single-carbon metabolism, and decreases in fatty acid oxidation. Increased reactive oxygen species (ROS) production and changes in the tricarboxylic acid cycle (TCA) intermediates may contribute to stabilizing hypoxia-inducible factors driving the observed metabolic changes. Arginine metabolism is related to NO production and the TCA cycle and plays a key role in the relationship between a vasoconstrictive phenotype and metabolic abnormalities in diseases [29]. In addition, the metabolism of alanine, aspartate, and glutamate is related to arginine biosynthesis [30]. The two pathways of arginine metabolism include the conversion of arginine to NO and the decomposition of arginine to urea and ornithine, which are regulated in the inflammatory response [31]. 

Serine/glycine metabolism is very important in biological metabolism because it provides one carbon unit, synthesizes other amino acids, nucleotides, and lipids, and synthesizes reducing substances to make REDOX in the cell in a state of dynamic equilibrium [32,33]. In recent years, studies have shown that serine/glycine metabolism plays a crucial role in tumors, affecting the occurrence and development of tumors and the therapeutic effect [34]. Metabolic remodeling can occur by promoting the proliferation of pulmonary artery smooth muscle cells (PASMCS), inducing pulmonary vascular remodeling, thus accelerating the development of PAH [35]. Metabolites are interrelated and mutually restricted. The change in one substance may be associated with the change in many substances, thus promoting the pathological process of PAH from different aspects. To elucidate the metabolic mechanism of AS in broilers by analyzing abnormal metabolic changes provides more targets for the prevention and control of AS.

In this study, in addition to DEGs significantly enriched in GO terms and KEGG pathways, we also found other important DEGs, *FABP4*, *APLNR*, *APLN*, *APELA*, *EIF2AK4*, *HMOX1*, *THBS1*, *TLR4*, *BCL2*, *FGF7*, *WNT5A*, and *CDK6*. The highly up-regulated differential gene, *FABP4*, also known as fatty acid binding protein 4, is mainly expressed in adipocytes and macrophages and is involved in regulating glucose and lipid metabolism related to inflammation and metabolic processes in target cells. FABP4 is associated with obesity, insulin resistance, diabetes mellitus, hypertension, cardiac dysfunction, atherosclerosis, and cardiovascular events [36,37]. FABP4 enhances hepatic glucose production in vivo and in vitro, reduces cardiomyocyte contraction in vitro, inhibits endothelial nitric oxide synthase (eNOS) expression/activation in vascular endothelial cells, increases vascular smooth muscle cell proliferation/migration and glucose-stimulated insulin secretion in pancreatic β-cells. FABP4 may also play a role in atherosclerosis through TLRs and other mechanisms [36,38]. Accumulating evidence for the pathogenic role of FABP4 in cardiovascular events suggests that FABP4 is a potential new target for preventing cardiovascular diseases. APLN is a novel peptide that is an endogenous ligand for the G protein-coupled receptor APLNR [39]. APLN–APLNR plays a key role in cardiac development, angiogenesis, NO-dependent vasodilation, and muscle strength signaling. APLN and APLNR are regulated by hypoxia, and the APLN–APLNR axis system has a protective effect on cardiovascular diseases [40]. APLN may inhibit the expression of fibroblast growth factor 2 (FGF2) and FGF receptor 1 (FGFR1) by regulating the expression of miR-424 and miR-503, thereby improving PAH [41]. In addition, APLN can regulate myocardial metabolism and glucose metabolism. Apelin treatment can substantially reduce the increase in myocardial free fatty acid (FFA) and glycogen content. In the treatment of type 2 diabetic rats, Apelin substantially reduced gene expression and increased the expression of CD36CPT-1 and PPAR-α in the heart fatty acid transporter [42]. APELA is also an endogenous ligand of APLNR, which is expressed in both lung and cardiac vascularization. Recent studies have shown that exogenous APELA and APLN have therapeutic significance for PAH in a monocrotaline rat model [43]. Additional studies have shown that APELA triggers vascular relaxation in a NO-independent manner, despite functional similarities between APELA and APLN. Therefore, like APLN, the effect of APELA on the vascular system needs further study [40]. EIF2AK4 expression leads to pulmonary vascular remodeling, pulmonary telangiectasia, a proliferation of vascular cells, and an increase in typical metabolites in oxidative stress, which are very important for the physiology and pathology of pulmonary hypertension [44]. HMOX1 is an inducible enzyme that can respond to a variety of stressors, including hypoxia, hyperoxia, acidosis, shear stress, and reactive oxygen species [45]. Additionally, it plays an important role in resolving pulmonary hypoxia and inflammation [46]. THBS1 is highly expressed in the plasma and pulmonary vessels of patients with pulmonary hypertension. It inhibits the production of NO through its cognate receptor, CD47 [47], and also participates in the pathogenesis of hypoxia-induced PAH through TGF β signaling [48]. TLR4 is expressed in platelets and mediates inflammation and the immune response [49]. Studies have shown that it plays an important role in maintaining normal pulmonary vasculature and the hypoxia-induced development of PAH [50,51]. BCL-2 can reduce cell apoptosis and improve cell activity by releasing cytochrome from mitochondria. Therefore, it is a valuable marker for PAH [52]. Wnts are secreted glycoproteins that control a variety of biological processes, especially cell proliferation, and trigger intracellular responses through various signaling pathways [53]. Recent studies have shown that the recruitment of classical and nonclassical Wnt pathways promotes the proliferation, survival, and migration of pulmonary arterial endothelial cells [54,55]. CDKs can bind to cyclin D1 to form complex phosphorylated substrates, which are involved in promoting cell cycle progression [56]. Studies have shown that JQ1+ reduces the expression of CDK2, CDK4, and CDK6 by increasing the expression of CDK inhibitor (CDKN) and CDKN2D to induce G1 cell cycle arrest. JQ1 + also inhibits the migration of serum stimulus pulmonary vascular endothelial cells (human pulmonary microvascular endothelial cell, HPMEC) [57]. These enriched genes were found to play an important role in broilers with AS and may be a way to explore a new intracellular signaling pathway in the pathogenesis of PAH, which is worthy of further study.

## 5. Conclusions

In summary, we successfully constructed a multifactor AS model and identified 1442 significant DGEs in the broiler lung using RNA-Seq technology. Thus, the lungs of AS-susceptible broilers may be a tissue in which the respiratory system is unable to deliver sufficient oxygen for metabolism. The results showed that the hypoxic stress response plays an important regulatory role and can initiate a large regulatory network of hypoxic stress. Key genes (*FABP4*, *APLNR*, *APLN*, *APELA*, *EIF2AK4*, *HMOX1*, *THBS1*, *TLR4*, *BCL2*, *FGF7*, *WNT5A*, and CDK6) play an important role in poultry AS, lung development, vascular remodeling and inflammation, and the metabolic process. Multiple pathways related to metabolic processes were found by KEGG enrichment, suggesting abnormal lung metabolism of AS in broilers. The genes identified in this study can be used as candidate genes for future lung studies of AS disease in broilers and hence provide a theoretical basis for studying AS in broilers.

## Figures and Tables

**Table 1:** Basic diet composition.

Dietary Composition	1–3 Weeks	4–6 Weeks
Corn (%)	54.6	58.2
Soybean meal (%)	33.0	29.5
Vegetable oil (%)	3.0	3.0
NaHCO_3_ (%)	1.5	1.3
Lime (%)	1.0	1.0
NaCl (%)	0.3	0.3
Lysine (%)	1.0	0.5
Methionine (%)	0.6	1.2
Premix (%)	5.0	5.0
Total (%)	100.0	100.0

**Table 2:** Basic diet nutrition level.

Nutrition Level	1–3 Weeks	4–6 Weeks
Metabolic energy (MJ/kg)	11.49	11.28
Crude protein (%)	≥21	≥21
Crude fat (%)	2.5	2.5
Crude fiber (%)	6	6
Crude ash content (%)	≤8	≤8
Calcium (%)	1.5	1.3
Total phosphorus (%)	0.6	0.6
Moisture content (%)	≤14	≤14
Vitamin A (IU)	1600	1600
Vitamin E (mg)	10	10
Vitamin D3 (IU)	220	220
Nicotinic acid (mg)	35	35
Pantothenic acid (mg)	12	12
Trace elements (mg)	30	30

**Table 3:** Forward and reverse primers used for qPCR validation of RNA-seq.

Primer	Forward Primer (5′ to 3′)	Reverse Primer(5′ to 3′)	Tm	GenBank
*WNT5A*	CAGCTCCGCTTGGATTACAGC	GGGTTCATGGGGTTCATAGGG	60	AB006014
*FABP4*	GCCTGACAAAATGTGCGACC	CTTCCTGGTAGCAAACCCCA	60	NM_204290
*FGF7*	GTGGCAATCAAAGGAGTGGA	AGTGGGATGCTCTGTGTTCTT	60	AB193566
*CDK6*	CCAGACCCGCACAACCTATT	ATGCGTTCACCTCACTGGAG	60	L77991
*APLNR*	TGCCTCAACCCCTTCCTCTA	TTTAGGTGCGGAAGAGCGTC	60	XM_001232340
*APELA*	AAGCTGCACCGACACAACTG	ACACTGAAAAGCCCGTTACGA	60	BG712694
*Gallus-β-actin*	GAGAGAAGATGACACAGATC	GTCCATCACAATACCAGTGG	60	L08165

**Table 4:** Effects of AS on growth performance, organ index in broilers.

	Treatment		Days		Treatment × Days	
Project Name	*F* Value	*p* Value	*F* Value	*p* Value	*F* Value	*p* Value
average body weight (g)	41.01	<0.001	220.19	<0.001	13.56	0.001
FCR (%)	0.001	0.97	1.66	0.231	0.76	0.486
AHI	57.85	<0.001	9.41	0.003	11.39	0.002
pulmonary organ coefficient (‰)	16.45	0.002	0.21	0.812	1.16	0.346

Note: Treatment includes normal group (N) and model group (C). Days refer to 15, 25, and 35 days old.

**Table 5:** Effect of broiler AS on blood routine parameters.

	Treatment		Days		Treatment × Days	
Project Name	*F* Value	*p* Value	*F* Value	*p* Value	*F* Value	*p* Value
RBC (10^12^/L)	88.16	<0.001	43.69	<0.001	21.87	<0.001
HB (g/L)	8.86	0.012	2.81	0.1	5.79	0.017
HCT (%)	21.42	0.001	0.87	0.443	7.04	0.009
PLT (10^9^/L)	1.76	0.208	8.76	0.005	4.81	0.029
PCT (%)	0.84	0.375	5.01	0.026	6.04	0.015
WBC (10^9^/L)	6.34	0.027	0.64	0.543	1.72	0.22
LYM (10^9^/L)	8.38	0.013	0.24	0.784	1.48	0.265
MID (10^9^/L)	3.16	0.101	0.74	0.495	2.96	0.09

Note: Treatment includes normal group (N) and model group (C). Days refer to 15, 25, and 35 days old.

**Table 6:** Top ten up-regulated and down-regulated genes in group C compared to group N.

Gene_id	Gene Name	*p*-Adjusted	Expression
ENSGALG00000015835	*GRIK1*	2.46019 × 10^−25^	Downregulated
ENSGALG00000010044	*KCNMB4*	4.82935 × 10^−21^	Downregulated
ENSGALG00000000284	*HOXB4*	2.072 × 10^−19^	Downregulated
ENSGALG00000014999	*MAP1B*	4.40191 × 10^−18^	Downregulated
ENSGALG00000009880	*INPP4B*	2.69568 × 10^−17^	Downregulated
ENSGALG00000050240	*TBX21*	6.28253 × 10^−16^	Downregulated
ENSGALG00000030969	*CLEC3B*	7.66948 × 10^−16^	Downregulated
ENSGALG00000028347	*SDCBP2*	8.79599 × 10^−16^	Downregulated
ENSGALG00000016266	*PRRG1*	1.02411 × 10^−15^	Downregulated
ENSGALG00000014692	*LMNB1*	2.43993 × 10^−14^	Downregulated
ENSGALG00000030025	*FABP4*	9.58707 × 10^−20^	Upregulated
ENSGALG00000012163	*BDNF*	3.10715 × 10^−19^	Upregulated
ENSGALG00000007304	*DNMBP*	5.25536 × 10^−18^	Upregulated
ENSGALG00000005552	*PTBP2*	6.28253 × 10^−16^	Upregulated
ENSGALG00000002638	*SRR*	4.3649 × 10^−15^	Upregulated
ENSGALG00000008439	*CD36*	5.54369 × 10^−14^	Upregulated
ENSGALG00000006563	*SEMA3D*	5.81136 × 10^−13^	Upregulated
ENSGALG00000012312	*GCAT*	8.31101 × 10^−13^	Upregulated
ENSGALG00000015107	*PIP5K1B*	1.02394 × 10^−12^	Upregulated
ENSGALG00000011035	*AK4*	1.77152 × 10^−11^	Upregulated

Note: GRIK1, glutamate ionotropic receptor kainate type subunit 1; KCNMB4, potassium calcium-activated channel subfamily M regulatory beta subunit 4; HOXB4, homeobox B4; MAP1B, microtubule associated protein 1B; INPP4B, inositol polyphosphate-4-phosphatase type II B; TBX21, T-box 21; CLEC3B, C-type lectin domain family 3 member B; SDCBP2, syndecan binding protein 2; PRRG1, proline rich and Gla domain 1; LMNB1, lamin B1; FABP4, fatty acid binding protein 4; BDNF, brain derived neurotrophic factor; DNMBP, dynamin binding protein; PTBP2, polypyrimidine tract binding protein 2; SRR, serine racemase-like; CD36, CD36 molecule; SEMA3D, semaphorin 3D; GCAT, glycine C-acetyltransferase; PIP5K1B, phosphatidylinositol-4-phosphate 5-kinase type 1 beta; AK4, adenylate kinase 4.

**Table 7:** Representative results of Gene Ontology and Kyoto Encyclopedia of Genes and Genomes (KEGG) pathway enrichment of differentially expressed genes.

Category	ID	Description	*p*-Value (*p* < 0.05)	Genes
GO term	GO:0048583	Regulation of response to stimulus	5.37 × 10^−10^	*EIF2AK4*, *HMOX1*, *MMP9*, *THBS1*, *NOS2*, *APLNR*, *APLN*, *AK4*, *CLEC3B*, *FABP4*, *DNMBP*, *AQP5*, *TLR4*, *CCR7*, *IGF-I*, *IGFBP*, *CD28*, *WNT5A*, *SSP1*, *FRZB*, *SFRP4*, *NR4A3*, *CAVIN4*
KEGG pathway	map04514	Cell adhesion molecules (CAMs)	1.93 × 10^−5^	*CD4*, *CD40*, *CD40LG*, *CD28*, *CD276*, *CD86*, *CD226*, *CD8BP*, *CD8A*, *CDH2*, *CDH15*, *SDC2*, *SDC4*, *ITGB7*, *ITGB8*, *ITGB2*, *ITGA8*, *ITGA9*, *OCLN*, *CLDN2*, *CLDN10*, *ICOSLG*, *ICOSLG*, *ICOS*, *PTPRF*, *PTPRC*, *NTNG1*, *NTNG2*, *NCAM1*, *NCAM2*, *NEO1*, *DMB2*, *BF2*, *LRRC4C*, *VCAN*, *YF5*, *BLB2*
KEGG pathway	map00520	Amino sugar and nucleotide sugar metabolism	0.008992198	*PGM3*, *HKDC1*, *GMDS*, *AMDHD2*, *GALT*, *CYB5R4*, *NANS*, *GNPDA2*, *GNPDA1*, *CYB5R2*, *PMM2*, *UAP1L1*, *UXS1*, *NPL*, *PMM1*, *CYB5RL*, *GFPT2*, *HK3*
KEGG pathway	map00250	Alanine, aspartate and glutamate metabolism	0.01445295	*ABAT*, *PPAT*, *GAD2*, *ADSS1*, *ASNS*, *GPT2*, *GLUL*, *ASPA*, *NAT8L*, *ASL1*, *RIMKLB*, *GFPT2*
KEGG pathway	map00260	Glycine, serine and threonine metabolism	0.017648565	*BPGM*, *MAOA*, *SRR*, *GCAT*, *DMGDH*, *SARDH*, *GATM*, *CHDH*
KEGG pathway	map04064	NF-kappa B signaling pathway	0.020172638	*ATM*, *TLR4*, *CD40*, *CD40LG*, *CCL4*, *CCL19*, *BCL2*, *BCL2L1*, *BCL2A1*, *TRIM25*, *TRAF1*, *PLCG1*, *PCASP2*, *PIAS4*, *PLCG2*, *PRKCB*, *RELA*, *CARD11*, *CSNK2A1*, *SH2D6*, *ZAP70*
KEGG pathway	map04066	HIF-1 signaling pathway	0.023554593	*CDKN1A*, *CDKN1B*, *TLR4*, *NOS2*, *ENO2*, *BCL2*, *IGF-I*, *HMOX1*, *HKDC1*, *HK3*, *EIF4EBP1*, *EGFR*, *RELA*, *PRKCB*, *PDK1*, *PLCG1*, *PLCG2*, *PFKFB3*, *TFRC*, *TF*, *ANGPT1*, *ANGPT4*, *CYBB*, *INSR*, *MKNK2*
KEGG pathway	map04115	p53 signaling pathway	0.023035501	*THBS1*, *BCL2*, *BCL2L1*, *CD82*, *CDK1*, *CDK6*, *CDKN1A*, *CASP8*, *IGF-1*, *IGFBP3*, *SESN1*, *SESN2*, *SESN3*, *STEAP3*, *ADGRB1*, *APAF1*, *ATM*, *RPRM*, *CHEK1*, *CHEK2*, *GADD45G*

## Data Availability

The research data in this sutdy has been deposited in NCBI SRA database with accession no. SUB9021659.

## References

[1] Gupta A.R. (2011). Ascites syndrome in poultry: A review. Worlds Poult. Sci. J..

[2] Fu X., Zhang F. (2018). Role of the HIF-1 signaling pathway in chronic obstructive pulmonary disease. Exp. Ther. Med..

[3] Lawson M., Jomova K., Poprac P., Kuča K., Musílek K., Valko M. (2018). Free radicals and antioxidants in human disease. Nutritional Antioxidant Therapies: Treatments and Perspectives.

[4] Stenmark K.R., Tuder R.M., El Kasmi K.C. (2015). Metabolic reprogramming and inflammation act in concert to control vascular remodeling in hypoxic pulmonary hypertension. J. Appl. Physiol..

[5] Davoodi P., Ehsani A. (2019). In-silico investigation of genomic regions related to ascites and identifying their pathways in broilers. Worlds Poult. Sci. J..

[6] Liu P., Yang F., Zhuang Y., Xiao Q., Cao H., Zhang C., Wang T., Lin H., Guo X., Hu G. (2017). Dysregulated expression of microRNAs and mRNAs in pulmonary artery remodeling in ascites syndrome in broiler chickens. Oncotarget.

[7] Sukumaran S., Jusko W.J., DuBois D.C., Almon R.R. (2011). Light-dark oscillations in the lung transcriptome: Implications for lung homeostasis, repair, metabolism, disease, and drug action. J. Appl. Physiol. (1985).

[8] Wang H., Huang P., Zegeng L.I., Zhao Z., Tong J., Yang C. (2017). Basis for water-liquid metabolism diseases from aspect of lung and ventilating lung qi for diuresis research. J. Changchun Univ. Chin. Med..

[9] Weiss D.J., Bertoncello I., Borok Z., Kim C., Panoskaltsis-Mortari A., Reynolds S., Rojas M., Stripp B., Warburton D., Prockop D.J. (2011). Stem cells and cell therapies in lung biology and lung diseases. Proc. Am. Thorac. Soc..

[10] Wang Y., Guo Y., Ning D., Peng Y., Yang Y., Liu D. (2013). Analysis of liver transcriptome in broilers with ascites and regulation by L-carnitine. J. Poult. Sci..

[11] Hasanpur K., Nassiri M., Hosseini Salekdeh G. (2019). The comparative analysis of phenotypic and whole transcriptome gene expression data of ascites susceptible versus ascites resistant chickens. Mol. Biol. Rep..

[12] Zhang J., Schmidt C.J., Lamont S.J. (2018). Distinct genes and pathways associated with transcriptome differences in early cardiac development between fast- and slow-growing broilers. PLoS ONE.

[13] Yang F., Cao H., Xiao Q., Guo X., Zhuang Y., Zhang C., Wang T., Lin H., Song Y., Hu G. (2016). Transcriptome analysis and gene identification in the pulmonary artery of broilers with ascites syndrome. PLoS ONE.

[14] Livak K., Schmittgen T. (2001). Analysis of relative gene expression data using real-time quantitative PCR and the 2(-Delta Delta C(T)) Method. Methods.

[15] Rao X., Huang X., Zhou Z., Lin X. (2013). An improvement of the 2ˆ(-delta delta CT) method for quantitative real-time polymerase chain reaction data analysis. Biostat. Bioinforma. Biomath..

[16] Currie R.J. (1999). Ascites in poultry: Recent investigations. Avian. Pathol..

[17] Özyİğİt M.Ö., Kahraman M.M., Akkoc A. (2015). The Effects of Hypoxia Expression of Vascular Endothelialgrowth Factor in Broiler Lung Fibroblasts. Turk. J. Vet. Anim. Sci..

[18] Tadzic R., Mihalj M., Vcev A., Ennen J., Tadzic A., Drenjancevic I. (2013). The effects of arterial blood pressure reduction on endocan and soluble endothelial cell adhesion molecules (CAMs) and CAMs ligands expression in hypertensive patients on Ca-channel blocker therapy. Kidney Blood. Press. Res..

[19] Chao J., Guo Y., Li P., Chao L. (2017). Opposing Effects of Oxygen Regulation on Kallistatin Expression: Kallistatin as a Novel Mediator of Oxygen-Induced HIF-1-eNOS-NO Pathway. Oxid Med Cell Longev..

[20] Jacquin S., Rincheval V., Mignotte B., Richard S., Humbert M., Mercier O., Londoño-Vallejo A., Fadel E., Eddahibi S. (2015). Inactivation of p53 is sufficient to induce development of pulmonary hypertension in rats. PLoS ONE.

[21] Liu J., Li J., Xie C., Xuan L., Tang B. (2020). MSCs attenuate hypoxia induced pulmonary hypertension by activating P53 and NF-kB signaling pathway through TNFα secretion. Biochem. Biophys. Res. Commun..

[22] Burke D.L., Frid M.G., Kunrath C.L., Karoor V., Anwar A., Wagner B.D., Strassheim D., Stenmark K.R. (2009). Sustained hypoxia promotes the development of a pulmonary artery-specific chronic inflammatory microenvironment. Am. J. Physiol. Lung. Cell. Mol. Physiol..

[23] Balabanian K., Foussat A., Dorfmüller P., Durand-Gasselin I., Capel F., Bouchet-Delbos L., Portier A., Marfaing-Koka A., Krzysiek R., Rimaniol A.C. (2002). CX_3_C chemokine fractalkine in pulmonary arterial hypertension. Am. J. Respir. Crit. Care Med..

[24] Semenza G.L. (2012). Hypoxia-inducible factors in physiology and medicine. Cell.

[25] Zhang J., Feng X., Zhao L., Wang W., Gao M., Wu B., Qiao J. (2013). Expression of hypoxia-inducible factor 1α mRNA in hearts and lungs of broiler chickens with ascites syndrome induced by excess salt in drinking water. Poult. Sci..

[26] Mézes M., Balogh K. (2011). Free radicals and antioxidants in avian diseases. Oxidative Stress in Applied Basic Research and Clinical Practice.

[27] Fessel J.P., Hamid R., Wittmann B.M., Robinson L.J., Blackwell T., Tada Y., Tanabe N., Tatsumi K., Hemnes A.R., West J.D. (2012). Metabolomic analysis of bone morphogenetic protein receptor type 2 mutations in human pulmonary endothelium reveals widespread metabolic reprogramming. Pulm. Circ..

[28] Peng B., Li H., Peng X.X. (2015). Functional metabolomics: From biomarker discovery to metabolome reprogramming. Protein Cell.

[29] Smolders V.F., Zodda E., Quax P.H.A., Carini M., Barberà J.A., Thomson T.M., Tura-Ceide O., Cascante M. (2018). Metabolic alterations in cardiopulmonary vascular dysfunction. Front. Mol. Biosci..

[30] Zhu W., Jiang X., Sun H., Li Y., Shi W., Zheng M., Liu D., Ma A., Feng X. (2021). Global lysine acetylation and 2-Hydroxyisobutyrylation profiling reveals the metabolism conversion mechanism in Giardia lamblia. Mol. Cell. Proteom..

[31] Qiu Y., Yang X., Wang L., Gao K., Jiang Z. (2019). L-arginine inhibited inflammatory response and oxidative stress induced by lipopolysaccharide via arginase-1 signaling in IPEC-J2 cells. Int. J. Mol. Sci..

[32] Padrón-Barthe L., Villalba-Orero M., Gómez-Salinero J.M., Acín-Pérez R., Cogliati S., López-Olañeta M., Ortiz-Sánchez P., Bonzón-Kulichenko E., Vázquez J., García-Pavía P. (2018). Activation of serine one-carbon metabolism by calcineurin Aβ1 reduces myocardial hypertrophy and improves ventricular function. J. Am. Coll. Cardiol..

[33] Martínez-Reyes I., Chandel N.S. (2014). Mitochondrial one-carbon metabolism maintains redox balance during hypoxia. Cancer Discov..

[34] Kit S. (1955). The biosynthesis of free glycine and serine by tumors. Cancer Res..

[35] Zhang R.Y., Liu J., Sun Y., Wang W., Wang C. (2022). Metabolic reprogramming in pulmonary hypertension. Zhonghua Jie He He Hu Xi Za Zhi.

[36] Girona J., Rosales R., Plana N., Saavedra P., Masana L., Vallvé J.C. (2013). FABP4 induces vascular smooth muscle cell proliferation and migration through a MAPK-dependent pathway. PLoS ONE.

[37] Ordovas J.M. (2007). Identification of a functional polymorphism at the adipose fatty acid binding protein gene (FABP4) and demonstration of its association with cardiovascular disease: A path to follow. Nutr. Rev..

[38] Furuhashi M. (2019). Fatty acid-binding protein 4 in cardiovascular and metabolic diseases. J. Atheroscler. Thromb..

[39] Yu X.H., Tang Z.B., Liu L.J., Qian H., Tang S.L., Zhang D.W., Tian G.P., Tang C.K. (2014). Apelin and its receptor APJ in cardiovascular diseases. Clin. Chim. Acta.

[40] Shin K., Kenward C., Rainey J.K. (2017). Apelinergic system structure and function. Compr. Physiol..

[41] Kim J., Kang Y., Kojima Y., Lighthouse J.K., Hu X., Aldred M.A., McLean D.L., Park H., Comhair S.A., Greif D.M. (2013). An endothelial apelin-FGF link mediated by miR-424 and miR-503 is disrupted in pulmonary arterial hypertension. Nat. Med..

[42] Feng J., Zhao H., Du M., Wu X. (2019). The effect of apelin-13 on pancreatic islet beta cell mass and myocardial fatty acid and glucose metabolism of experimental type 2 diabetic rats. Peptides.

[43] Frump A.L., Bonnet S., de Jesus Perez V.A., Lahm T. (2018). Emerging role of angiogenesis in adaptive and maladaptive right ventricular remodeling in pulmonary hypertension. Am. J. Physiol. Lung Cell Mol. Physiol..

[44] Tenorio J., Navas P., Barrios E., Fernández L., Nevado J., Quezada C.A., López-Meseguer M., Arias P., Mena R., Lobo J.L. (2015). A founder EIF2AK4 mutation causes an aggressive form of pulmonary arterial hypertension in Iberian Gypsies. Clin. Genet..

[45] Liang O.D., Mitsialis S.A., Chang M.S., Vergadi E., Lee C., Aslam M., Fernandez-Gonzalez A., Liu X., Baveja R., Kourembanas S. (2011). Mesenchymal stromal cells expressing heme oxygenase-1 reverse pulmonary hypertension. Stem Cells.

[46] Zhou L., Wang L.M., Song H.M., Shen Y.Q., Xu W.J., Xu J.H., Liu Y., Yan W.W., Jiang J.F. (2013). Expression profiling analysis of hypoxic pulmonary disease. Genet. Mol. Res..

[47] Jacob S.A., Novelli E.M., Isenberg J.S., Garrett M.E., Chu Y., Soldano K., Ataga K.I., Telen M.J., Ashley-Koch A., Gladwin M.T. (2017). Thrombospondin-1 gene polymorphism is associated with estimated pulmonary artery pressure in patients with sickle cell anemia. Am. J. Hematol..

[48] Kumar R., Mickael C., Kassa B., Sanders L., Hernandez-Saavedra D., Koyanagi D.E., Kumar S., Pugliese S.C., Thomas S., McClendon J. (2020). Interstitial macrophage-derived thrombospondin-1 contributes to hypoxia-induced pulmonary hypertension. Cardiovasc. Res..

[49] Sun Z. (2014). Platelet TLR4: A critical link in pulmonary arterial hypertension. Circ. Res..

[50] Ma L., Chang J., Wu H., Chen Y. (2011). Hypoxia-induced pulmonary arterial hypertension: The role of TLR4. Blood.

[51] Ma L., Ambalavanan N., Liu H., Sun Y., Jhala N., Bradley W.E., Dell’Italia L.J., Michalek S., Wu H., Steele C. (2016). TLR4 regulates pulmonary vascular homeostasis and remodeling via redox signaling. Front. Biosci. Landmark Ed..

[52] Benza R.L., Williams G., Wu C., Shields K.J., Raina A., Murali S., Passineau M. (2016). In situ expression of Bcl-2 in pulmonary artery endothelial cells associates with pulmonary arterial hypertension relative to heart failure with preserved ejection fraction. Pulm. Circ..

[53] Kang Z., Ji Y., Zhang G., Qu Y., Zhang L., Jiang W. (2016). Ponatinib attenuates experimental pulmonary arterial hypertension by modulating Wnt signaling and vasohibin-2/vasohibin-1. Life Sci..

[54] Sklepkiewicz P., Schermuly R.T., Tian X., Ghofrani H.A., Weissmann N., Sedding D., Kashour T., Seeger W., Grimminger F., Pullamsetti S. (2011). Glycogen synthase kinase 3beta contributes to proliferation of arterial smooth muscle cells in pulmonary hypertension. PLoS ONE.

[55] Takahashi J., Orcholski M., Yuan K., de Jesus Perez V. (2016). PDGF-dependent β-catenin activation is associated with abnormal pulmonary artery smooth muscle cell proliferation in pulmonary arterial hypertension. FEBS Lett..

[56] Cui C., Zhang H., Guo L.N., Zhang X., Meng L., Pan X., Wei Y. (2016). Inhibitory effect of NBL1 on PDGF-BB-induced human PASMC proliferation through blockade of PDGFβ-p38MAPK pathway. Biosci. Rep..

[57] Mumby S., Gambaryan N., Meng C., Perros F., Humbert M., Wort S.J., Adcock I. (2017). Bromodomain and extra-terminal protein mimic JQ1 decreases inflammation in human vascular endothelial cells: Implications for pulmonary arterial hypertension. Respirology.

